# Non linear dynamics of mechanosensory flight control in flies

**DOI:** 10.1186/1471-2202-14-S1-F3

**Published:** 2013-07-08

**Authors:** Martin Zapotocky, Jan Bartussek, Steven N Fry

**Affiliations:** 1 Institute of Physiology, Academy of Sciences of the Czech Republic, Prague 14220, Czech Republic; 2 Institute of Neuroinformatics, University of Zurich and ETH Zurich, Zurich CH-8057, Switzerland; 3 SciTrackS GmbH, CH-8118 Pfaffhausen, Switzerland

## 

In most animals, rhythmic motion is governed by central pattern generators (CPGs) - neural circuits that generate periodic patterned output. Sensory input to the CPG is not necessary to maintain the periodic neural activity, but is known to strongly influence it. For example, proprioceptive afferent input may entrain the neural CPG rhythm to the mechanical resonance frequency of a limb [1]. CPGs have been shown to govern the locomotor rhythms in vertebrates as well as in insects. A CPG governing the flight rhythm, however, has not been identified in flies, which are known for their remarkable flight maneuverability. In flies, the wingbeat rhythm is generated myogenically by stretch-activated power muscles and hence is not directly controlled by neural input. It is therefore unclear if the insights gained for proprioceptive feedback in CPG-based motor systems [1] likewise apply to flight control in flies.

In our study, we investigated if and how proprioceptive feedback influences the rhythm of the myogenic wing beat oscillator. We concentrated on mechanosensory input from the halteres - specialized „gyroscopic" sensory organs of flies. The halteres are known to activate the motor neurons of miniscule steering muscles, which in turn modulate the motion of the wings. In our experiments [2], tethered flying fruit flies (*Drosophila melanogaster*) were vibrated by a piezoelectric actuator to stimulate the haltere mechanosensory pathways. We used a laser Doppler interferometer to measure the vibrations of the tether resulting from the superposition of the piezo-delivered stimulus and the fly's wing beat. We determined the phase relationship between the wing motion and the mechanical stimulus in each wing stroke and applied an automated synchrogram analysis [3] to detect entrainment and higher-order synchronization. The flies synchronized with the stimulus for specific ranges of stimulus amplitude and frequency, revealing the characteristic Arnol'd tongues of a forced limit cycle oscillator. Our analysis shows that the steering muscles (activated by proprioceptive input) act as a *mechanical *forcing of the central power muscle oscillator. We propose that the mechanical forcing of a myogenic limit cycle oscillator permits flies to avoid the comparatively slow control based on a neural central pattern generator.

In the entrainment study described above, flies were attached to tethers with high resonance frequency and damping coefficient. While experimenting with tethers of varying mechanical properties, however, we observed that the fly's behavior was significantly influenced by the properties of the tether. To systematically explore this influence for a given fly, we altered the tether's resonance frequency by clamping the tether at a different point in each distinct flight test. In these experiments, no piezo stimulus was applied. Yet, when the tether resonance frequency was comparable to the wing beat frequency, the forces from the fly lead to large cumulative motion of the tether and activation of the haltere mechanosensors. The fly therefore received delayed feedback dependent on its previous activity. This lead to a variety of observed dynamical regimes, including the locking of the wing beat frequency to the tether resonance frequency when their initial difference was sufficiently small (similar to limb entrainment in [1]). We were able to reproduce most of the observed dynamical features in a simplified mathematical model of two mutually coupled oscillators: a phase-reduced nonlinear oscillator representing the wing beat and a linear oscillator representing the tether dynamics.

## References

[B1] Hatsopoulos NG Coupling the neural and physical dynamics in rhythmic movements Neural Computation 1996 8 567 581 10.1162/neco.1996.8.3.567 8868568

[B2] Bartussek J Mutlu AK Zapotocky M Fry SN Limit-cycle-based control of the myogenic wingbeat rhythm in the fruit fly *Drosophila* Journal of the Royal Society Interface 2013 10 20121013 10.1098/rsif.2012.1013 PMC3565748 23282849

[B3] Hamann C Bartsch RP Schumann AY Penzel T Havlin S Kantelhardt JW Automated synchrogram analysis applied to heartbeat and reconstructed respiration Chaos 2009 19 015106 10.1063/1.3096415 19335010

